# Adding droxidopa to fludrocortisone or midodrine in a patient with neurogenic orthostatic hypotension and Parkinson disease

**DOI:** 10.1007/s10286-017-0434-6

**Published:** 2017-07-03

**Authors:** Daniel Kremens, Mark Lew, Daniel Claassen, Brent P. Goodman

**Affiliations:** 1 Department of Neurology, Thomas Jefferson University, Philadelphia, PA USA; 2 Department of Neurology, University of Southern California, Los Angeles, CA USA; 3 Department of Neurology, Vanderbilt University, Nashville, TN USA; 4 Department of Neurology, Mayo Clinic Arizona, Scottsdale, AZ USA

**Keywords:** Fludrocortisone, Midodrine, Droxidopa, Neurogenic orthostatic hypotension, Parkinson’s disease

## Challenge questions

Is it appropriate to add droxidopa in a patient already taking fludrocortisone or midodrine? Should droxidopa substitute any of the previous treatments?

## Case presentation

MP is a 60-year-old female who was diagnosed with Parkinson disease (PD) 6 years ago. She was started on one tablet of carbidopa/levodopa 25/100 mg three times daily (TID), in addition to carbidopa 25 mg with each dose of carbidopa/levodopa, to enhance the conversion from levodopa to dopamine in the central nervous system. Last year, her carbidopa/levodopa dosage was increased to 1.5 tablet TID, and entacapone 200 mg TID was added. Over the past 6 months, she has developed marked episodes of lightheadedness upon standing.

She was evaluated by her primary care physician and diagnosed with orthostatic hypotension (OH). The doctor instructed her to use non-pharmacologic measures to help relieve her symptoms of OH, including increasing fluids, liberalizing salt intake, and wearing compression stockings. Despite these measures, she remained symptomatic so her primary care physician began treatment with fludrocortisone 0.1 mg once a day. The doctor recently increased the dose to 0.2 mg, but she still had little improvement in her symptoms of lightheadedness and dizziness upon standing.

MP then saw her neurologist, and it was noted that, despite the treatment with fludrocortisone, she remained symptomatic. Office blood pressure (BP) and heart rate (HR) measurements showed that her supine BP was 178/99 mmHg with a HR of 78 beats per minute (bpm), and 3 min after standing her BP was 91/62 mmHg with an HR of 80 bpm, and she felt lightheaded.

Due to her diagnosis of PD and her BP measurements, the patient was diagnosed with neurogenic OH (nOH). She remained symptomatic despite treatment with non-pharmacologic measures of liberalizing fluids, wearing compression stockings up to the abdomen, salting her food, and receiving fludrocortisone 0.2 mg/day.

## Expert commentary (Dr. Kremens)

Fludrocortisone is a synthetic mineralocorticoid that is sometimes used off-label to treat nOH. Its effects to increase BP are due to sodium and water retention, thereby increasing circulating blood volume. Fludrocortisone also causes hypokalemia and can cause or aggravate renal failure. In an elderly population, concern for fluid overload leading to congestive heart failure needs to be considered. It does not, however, have any effect on the blunted norepinephrine (NE) release from post-ganglionic sympathetic nerves upon standing, which is the underlying pathophysiologic mechanism causing nOH.

## Case continuation

It was decided that it would be appropriate to treat MP with droxidopa, an oral synthetic precursor of NE, which acts in both the central and peripheral nervous system and is thought to improve nOH by replenishing plasma NE levels [1, 4]. MP was started on droxidopa at 100 mg on a modified TID schedule (taken prior to arising from bed in the morning, at midday, and the last dose at least 3–4 h before bedtime to limit the potential for supine hypertension, which is a frequent complication in patients with PD and OH [2, 6]). She was instructed to up-titrate the droxidopa dose by 100 mg every 24–48 h until she felt symptomatically improved or until she had reached the maximum dosage of 600 mg TID, whichever occurred first. While titrating droxidopa, the patient was instructed to measure her supine BP readings 1 h after taking the droxidopa initially and after each dose increase to evaluate for supine hypertension. The patient also continued taking fludrocortisone 0.2 mg/day after adding droxidopa. Although MP did not substantially improve on fludrocortisone, she did not experience any adverse effects. It was therefore continued. Potential reasons for withdrawing the fludrocortisone would include adverse side effects such as supine hypertension, ankle edema, or hypokalemia.

## Expert commentary (Dr. Lew)

It would make sense to continue fludrocortisone as the patient is not experiencing side effects with this drug. Adding droxidopa, a precursor to NE, was subsequently warranted. In one of the phase III clinical trials of droxidopa for nOH [3], approximately one-third of patients had droxidopa added to fludrocortisone successfully. The patient should also be reminded to take her droxidopa approximately at the same time each day and not to take it within 4 h of a nap or sleeping. Another conservative measure the patient should be counseled to undertake would be to elevate the head of her bed at least 30–45° at night when sleeping; this would diminish the risk for ongoing supine hypertension, reduce nocturnal natriuresis, and improve morning BP.

## Expert commentary (Dr. Goodman)

Fludrocortisone is most helpful in patients who are unable to expand blood volume by increasing salt and fluid intake. This most frequently occurs in individuals with gastrointestinal problems (such as chronic diarrhea), which results in volume depletion, and in the elderly, who may be reluctant to drink extra fluids because doing so requires a lifestyle adjustment or may lead to an increase in urination. In my experience, increasing the dose of fludrocortisone beyond 0.2 mg per day does not benefit OH and is more likely to result in hypokalemia and other adverse events.

## Expert commentary (Dr. Claassen)

For this patient, when in the course of treatment with droxidopa, I would also consider adding midodrine, although no dedicated studies combining both droxidopa and midodrine have been performed so far. Midodrine is a short-acting alpha-2 agonist and works effectively to increase BP and improve symptoms of nOH [5]. If a patient needs to stand for predictable short periods of time, it is reasonable to add midodrine to the regimen to improve orthostatic tolerance. Note that midodrine does not cross the blood-brain barrier, and its use, as with fludrocortisone and droxidopa, is associated with supine hypertension. Using midodrine “as needed” remains a valuable tool for acute needs of BP control. In addition, I agree that fludrocortisone is a useful medication that effectively increases blood volume. In this manner, if this patient is experiencing low blood volume, it can work in combination with droxidopa and/or midodrine.

## Case continuation

The patient achieved a droxidopa dosage of 600 mg TID and continued treatment with fludrocortisone 0.2 mg/day. However, she continued to experience symptoms of nOH, particularly lightheadedness and dizziness in the morning. Her supine BP was 179/102 mmHg with a heart rate of 81 bpm. After 3 min of standing, her BP was 90/54 mmHg with a heart rate of 89 bpm.

## Expert commentary (Dr. Claassen)

In addition to potentially adding midodrine in this patient, I would also consider the enzymatic breakdown of droxidopa. The addition of carbidopa may reduce the peripheral breakdown of droxidopa. Droxidopa is metabolized to its active form by the enzyme dopa-decarboxylase, which is inhibited by carbidopa outside the CNS. While phase 3 clinical trials assessing droxidopa failed to show a strong relationship between carbidopa and the need for higher doses of droxidopa for efficacy [4], it is worthwhile considering this potential interaction. Furthermore, entacapone, a catechol-O-methyl transferase (COMT) inhibitor, may also augment the pharmacokinetic breakdown of droxidopa. In this case, the main metabolite of droxidopa, 3-*O*-methyl-DOPS, is metabolized by COMT, such that a COMT inhibitor may potentiate the effect of droxidopa. For this reason, a clinician may desire to increase the morning dose of droxidopa to 900 mg and test whether improved efficacy of symptoms is noted. It is necessary to point out that 900 mg of droxidopa is a higher dose than in the approved labeling; therefore, tolerability and safety have not been studied at this dose level.

## Case conclusion

Due to continued nOH symptoms in the morning, the patient was prescribed midodrine 5 mg BID as needed. The patient was asked to monitor her BP 1 h after midodrine administration to ensure that her BP was not too high with this regimen. The addition of midodrine as needed in the morning, along with continued use of droxidopa and fludrocortisone, relieved her morning nOH symptoms.
